# Aggregation of *p*-Sulfonatocalixarene-Based Amphiphiles and Supra-Amphiphiles

**DOI:** 10.3390/ijms14023140

**Published:** 2013-02-04

**Authors:** Nuno Basilio, Vitor Francisco, Luis Garcia-Rio

**Affiliations:** Physical Chemistry Department, Center for Research in Biological Chemistry and Molecular Materials (CIQUS), University of Santiago, 15782 Santiago, Spain; E-Mails: nuno.basilio@usc.es (N.B.); francisco.vms@gmail.com (V.F.)

**Keywords:** calixarenes, surfactants, supramolecular amphiphiles, micelle, vesicle

## Abstract

*p*-Sulfonatocalixarenes are a special class of water soluble macrocyclic molecules made of 4-hydroxybenzenesulfonate units linked by methylene bridges. One of the main features of these compounds relies on their ability to form inclusion complexes with cationic and neutral species. This feature, together with their water solubility and apparent biological compatibility, had enabled them to emerge as one the most important host receptors in supramolecular chemistry. Attachment of hydrophobic alkyl chains to these compounds leads to the formation of macrocyclic host molecules with amphiphilic properties. Like other oligomeric surfactants, these compounds present improved performance with respect to their monomeric counterparts. In addition, they hold their recognition abilities and present several structural features that depend on the size of the macrocycle and on the length of the alkyl chain, such as preorganization, flexibility and adopted conformations, which make these molecules very interesting to study structure-aggregation relationships. Moreover, the recognition abilities of *p*-sulfonatocalixarenes enable them to be applied in the design of amphiphiles constructed from non-covalent, rather than covalent, bonds (supramolecular amphiphiles). In this review, we summarize the developments made on the design and synthesis of *p*-sulfonatocalixarenes-based surfactants, the characterization of their self-assembly properties and on how their structure affects these properties.

## 1. Introduction

**Figure 1:**
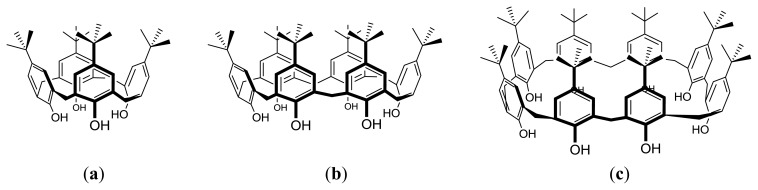
Structures of *tert*-butylcalix[*n*]arenes. (**a**) *n* = 4; (**b**) *n* = 6; (**c**) *n* = 8.

Calix[*n*]arenes are macrocyclic oligomers made of *n* phenol units linked by methylene bridges at the *ortho* position [1,2]. These compounds result from the condensation of *para*-substituted (usually *tert*-butyl groups) phenols with formaldehyde. Depending on the reaction conditions, calixarenes with four up to 20 phenol units can be obtained [3], though those with four, six and eight units are the most common (Figure 1). The popularity of calixarene building blocks in supramolecular chemistry can be attributed, at least partially, to their facile chemical functionalization. After removal of the *tert*-butyl groups from the calixarene framework using AlCl_3_ in dry toluene, they can be readily and selectively functionalized at the hydroxyl groups (lower rim) and at the *para* positions (upper rim), owing to the different chemical reactivity of these groups [2]. Selective functionalization of the calixarene rims can be used to enhance their receptor ability and selectivity towards target guests or to develop sophisticated functional applications, such as rotaxanes and catenanes [4–9], nanoparticles and monolayers [10,11], sensors for enzyme assays [12], drug delivery systems [13], supramolecular polymers [14] and surfactants [15].

In addition to the diverse calixarene structures that can be devised by varying the size of the macrocycle and the nature of the substituents, their characteristic conformational isomerism can also contribute to further increase the diversity of functional molecules that can be constructed from the calixarene framework. While in Figure 1 all three calixarenes are depicted in the so-called cone conformation, the phenol units can undergo rotation through the annulus, yielding other conformational isomers. In the case of calix [4]arene derivatives, four main conformations are possible: cone, partial cone, 1,2-alternate and 1,3-alternate; while calix [6] and calix [8]arenes can display eight and sixteen main conformations, respectively [1,16]. It should be noted that besides these “up-down” conformations, several others in which the aryl rings adopt planar orientations may be possible.

Among the various types of calixarenes described in the literature, *p*-sulfonatocalix[*n*]arenes (SCn) had attracted considerable attention due to their high water solubility, selective binding ability, catalytic properties and apparent biological compatibility [17–24]. In fact, several biological and medical potential applications that take advantage of the SCn properties have been described [25]. In addition to these interesting properties, *p*-sulfonatocalix[*n*]arenes can also be used as amphiphiles (or surfactants) that are able to self-assemble into well-defined nanostructures, such as micelles, when placed in aqueous solution. *p*-sulfonatocalixarene-based amphiphiles can be simply obtained by attaching hydrophobic alkyl chains to the phenolic oxygen atoms. These compounds can be, in principle, included in the same category of oligomeric surfactants (such as gemini (dimeric), trimeric and tetrameric surfactants) [26–36]. Besides sharing some of the appellative properties of open chain oligomeric surfactant, such as lower critical micelle concentration (CMC), calixarene-based surfactants are equipped with a host-guest recognition site, able to bind guest molecules and ions, which can be explored along with their self-association properties in order to develop new soft materials.

In addition to the synthesis of SCn-based surfactants by using irreversible covalent bonds, the recognition properties of SCn enable them to be also used as building blocks to construct supramolecular amphiphiles (or supra-amphiphiles). In these cases, the functional segments required to provide or improve the amphiphilic properties of the molecular species are linked by means of non-covalent interactions [37]. These amphiphiles can be classified by the type of complex formed between the independent molecular species that are involved in their composition. In that sense, supra-amphiphiles comprising calixarenes or other macrocyclic receptors, such as cucurbiturils and cyclodextrins, can be placed in the host-guest category. In addition to host-guest complexes, charge-transfer, hydrogen bonded, ionic or coordination complexes, among others, with amphiphilic properties have been described and recently reviewed [37].

The supramolecular approach is not only effective, but also capable of making the synthetic process simpler and highly reversible when compared with covalently bonded systems. The reversibility is in fact a key feature of these systems, since it allows the assembly process to be controlled by external stimuli and, thus, enables supra-amphiphiles to be applied as smart materials. In the following review, we will try to provide an overview of the work published on the aggregation of SCn-based amphiphiles and supra-amphiphiles.

## 2. Amphiphilic Sulfonatocalixarenes: Structure-Aggregation Relationships

### 2.1. Earlier Work

**Figure 2:**
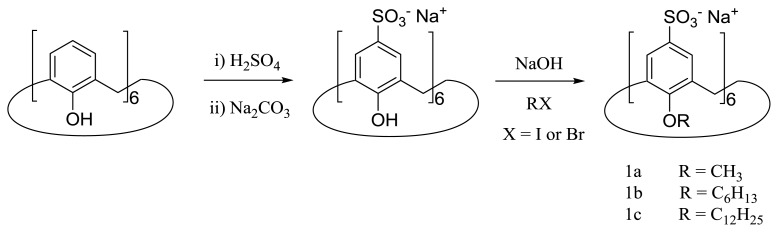
Synthesis of alkylated *p*-sulfonatocalix [6]arenes.

The first example of calixarenes with surface active properties was reported by Shinkai *et al.* back in 1986 [38,39]. In this pioneering work, the authors described the synthesis of *p*-sulfonatocalix [6]arenes bearing alkyl chains at the lower rim. These compounds were obtained by sulfonation of the parent calix [6]arene with H_2_SO_4_, followed by neutralization and alkylation with alkyl halides in basic media (Figure 2). It was found that compound 1b forms small spherical micelles at a concentration above the CMC = 0.6 mM, with an aggregation number (*N*) of six or 19 unimers per micelle if determined by light scattering or estimated from the solubilization test of orange OT (1-(*o*-Tolylazo)-2-naphthol), respectively. On the other hand, for the **1c** derivative, some unexpected properties, such as the absence of a clear CMC, surface inactivity, *N* values of about two and formation of 1:1 host guest complexes with small dye molecules were reported. Based on these results, it was proposed that **1c** rather acts as a “unimolecular” micelle. A small angle x-ray scattering study of these compounds afforded values of *N* for **1b** compatible with that previously reported by Shinkai *et al.*, but the results obtained for **1c** are substantially different [40]. Specifically, the authors reported very high *N* values ranging from 84 to 217, depending on the concentration and on the assumptions made for the estimation of this parameter. Nevertheless, these results are obviously inconsistent with the proposed unimolecular micelle.

**Figure 3:**
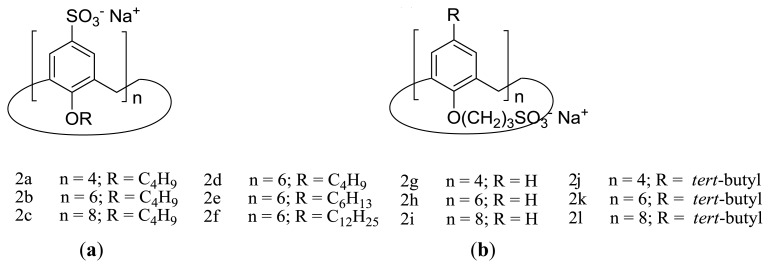
Amphiphilic sulfonatocalix[*n*]arenes bearing the hydrophilic SO_3_^−^ group at the (**a**) upper rim and (**b**) lower rim.

To complement their work on the self-assembly of amphiphilic sulfonatocalixarenes, the Shinkai group synthesized a new collection of derivatives (Figure 3) and discussed possible structure-aggregation relationships [41,42]. Based on the ^1^H NMR pattern of signals, it was possible to verify that the calix [4]arenes derivatives (**2a**, **2g** and **2j**) adopt a fixed cone conformation, while all amphiphilic calix [6]arenes and calix [8]arenes studied exchange between several possible conformations, due to the unhindered rotation of the phenolic units through the annulus.

The CMC’s of these series of compounds were determined by conductance and surface tension measurements. The results obtained allowed the authors to propose a classification based on the aggregation properties of these types of calixarene derivatives. Accordingly, calixarenes that do not bear long alkyl chains (e.g., **1a**, **2g**–**i**) do not form micelles and, thus, are classified as (i) nonmicellar calixarenes. On the other hand, calixarenes with moderate alkyl chain lengths (e.g., **1b**, **2a**–**e** and **2j**–**l**) self-assemble into micelles and are classified as (ii) micelle-forming calixarenes, while those with long alkyl chains (e.g., **1c** and **2f**) were placed in the category of (iii) unimolecular micelle calixarenes.

It was also observed that the ^1^H NMR chemicals shifts for some protons of **2a** are displaced to a higher magnetic field when the concentration is increased above the CMC [42]. It was reported that the signals corresponding to the *endo* protons of the methylene bridge and to the OCH_2_ protons of the alkyl chains were particularly affected, while, on the other hand, those corresponding to the aromatic protons hardly changed. By considering these observations, a model was proposed for the micellar aggregates in which the alkyl chains form the micellar core and the aromatic rings form stacks at the surface of the micelle. Results obtained from fluorescence polarization experiments suggested that the microviscosity of the calixarene-based micelles is generally higher than that of conventional surfactant micelles.

### 2.2. Conformational Reorganization upon Micellization

After the seminal work conducted by the Shinkai research group on the synthesis and aggregation of amphiphilic *p*-sulfonatocalix[*n*]arenes, these molecules have been sporadically revisited by several researchers, but, to the best of our knowledge, none of these works were focused on the aggregation properties of this special class of surfactants [43–58]. Recently, our group has decided to undertake a systematic study on the self-assembly of such amphiphiles [59–61]. The motivation for this work was related to some physical characteristics of the micellar systems formed from calixarene-based surfactants, such as slow unimer-micelle exchange rates and low CMC’s, which are not fully understood [59,62,63].

**Figure 4:**
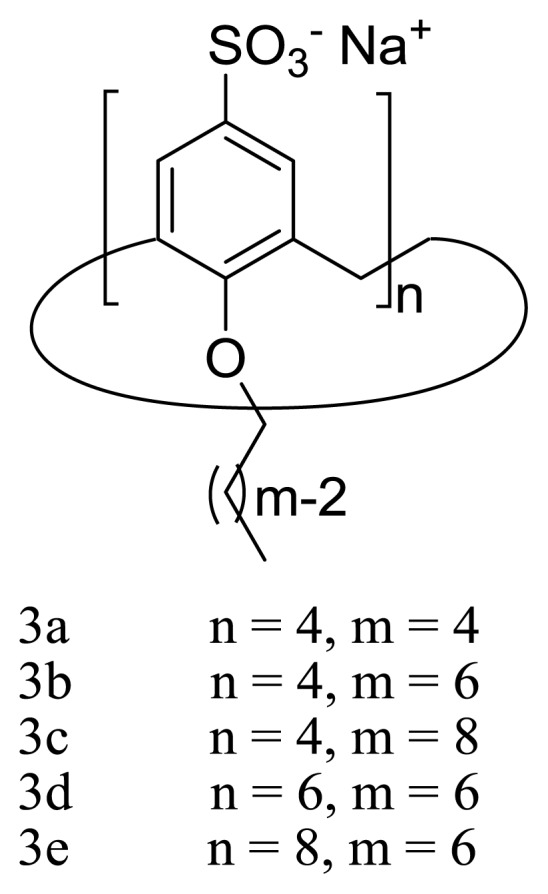
Amphiphilic *p*-sulfonatocalix[*n*]arenes used to establish structure aggregation relationships in references [60,61].

**Figure 5:**
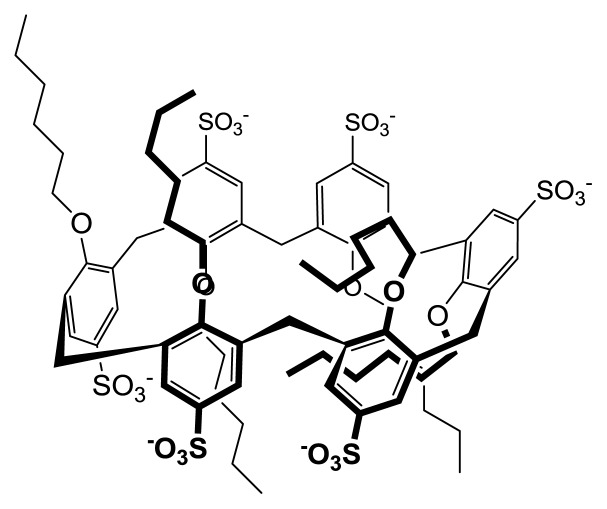
Pseudo 1,2,3-alternate conformation adopted by calix [6]arene **3d** below the CMC.

In the first instance, it was decided to investigate the micellization of three calix [4]arene derivatives with alkyl chains of different lengths at the lower rim together with a calix [6]arene and a calix [8]arene, both bearing alkyl chains of the same length (hexyl) at the lower rim (Figure 4). With these five compounds, it is possible to establish correlations between the micellization properties with both the alkyl chain length and the number of phenolic units present in the macrocyclic ring (*i.e*., the ring size). While all three calix [4]arene-based surfactants (**3a**–**c**) were found to be, as expected, blocked into the cone conformation, the hexamer **3d** adopts a pseudo-1,2,3-alternate conformation at concentrations below the CMC (Figure 5). It must be noted that **3d** is not physically blocked into this conformation, but stabilized, presumably, by hydrophobic intramolecular interactions [61]. On the other hand, the octamer **3e** seems to present a loose structure, and its ^1^H NMR spectra indicates that, below the CMC, the molecule undergoes fast exchange between several possible conformers.

**Figure 6:**
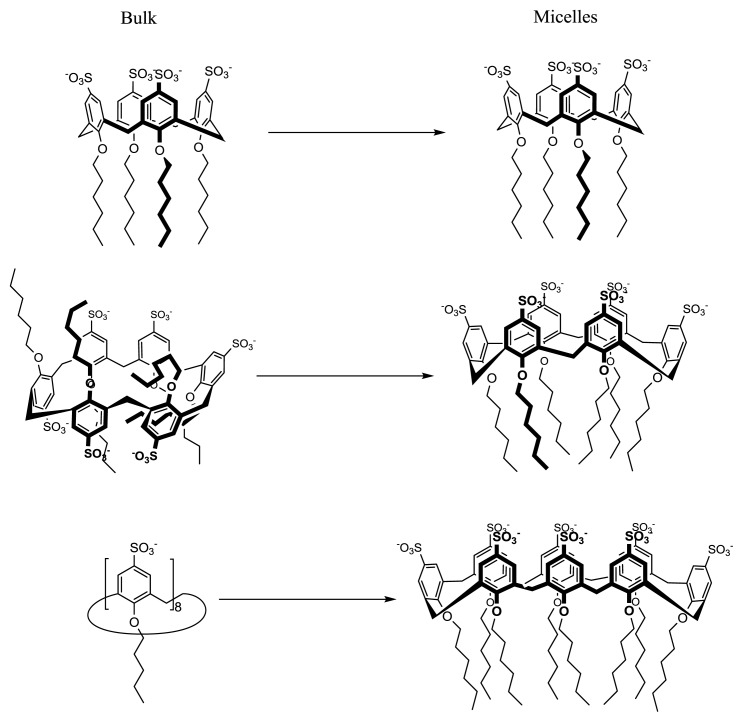
Conformational reorganization of amphiphilic *p*-sulfonatocalix[*n*]arenes upon micellization.

By increasing the concentration of amphiphilic *p*-sulfonatocalix[*n*]arenes to values above the CMC, it was observed that these surfactants can undergo a structural reorganization upon aggregation to adopt the cone conformation in the micelles (Figure 6). This conformational change, induced by the aggregation process, demonstrates that the cone conformation is stabilized in the micelles. This result was expected, since in the micelles, the alkyl chains should point to the hydrophobic interior, while the sulfonate groups remain in contact with the solvent [61]. In the same article, it was also reported that the micelle diffusion coefficients obtained from diffusion ordered NMR spectroscopy (DOSY) experiments. From these results, it was suggested that the micellar assemblies formed from amphiphilic *p*-sulfonatocalix[*n*]arenes well above the CMC adopt ellipsoidal, rather than spherical, geometries.

### 2.3. Thermodynamics of Micellization

The CMC’s of the *p*-sulfatocalix[*n*]arenes-based surfactants depicted in Figure 4 were determined by conductance [61]. The obtained data shows that the log (CMC) decreases linearly with the number of carbons present in the alkyl chains, suggesting the hydrophobic interactions contribute favorably to the micellization process. On the other hand, when the values of log (CMC) are correlated with the number of monomeric units present in the macrocyclic structure, it is observed that the tendency to self-aggregate into micelles decreases with this parameter. While the first correlation is in line with the generally observed behavior for conventional surfactants, the increase in the CMC (in mole of alkyl chain units) with the number of monomeric units is contrary to what was expected on the basis of the results reported for oligomeric surfactants [26–36]. To get more insight into this particular observation, the micellization of these compounds was further studied by isothermal titration calorimetry (ITC) [60].

The results obtained in this work show that, in the interval ranging from 288 to 328 K, all calixarenes investigated present negative values for the enthalpy of micellization (Δ*H*_M_), and it became more exothermic with increasing the temperature. This dependence is characteristic of processes where the hydrophobic effect plays a significant role. Besides Δ*H*_M_, the free energy of micellization (Δ*G*_M_) can also be determined, and consequently, knowing these two thermodynamic functions, the entropy of micellization (Δ*S*_M_) can be also calculated. As can be easily demonstrated, Δ*G*_M_ (in kJ·mol^−1^ of alkyl chain) is given by Equation 1 [60,64]:

(1) $$\Delta G_{M}=RT\left(\frac{1}{j}+\beta \right)\text{ln }CMC-RT\frac{\text{ln }j}{j}$$

where *j* is the number of alkyl chains, *R* is the ideal gas constant, *T* is the temperature, β is the fraction of neutralized micellar charge and the CMC is expressed in mole of alkyl chain units.

**Figure 7:**
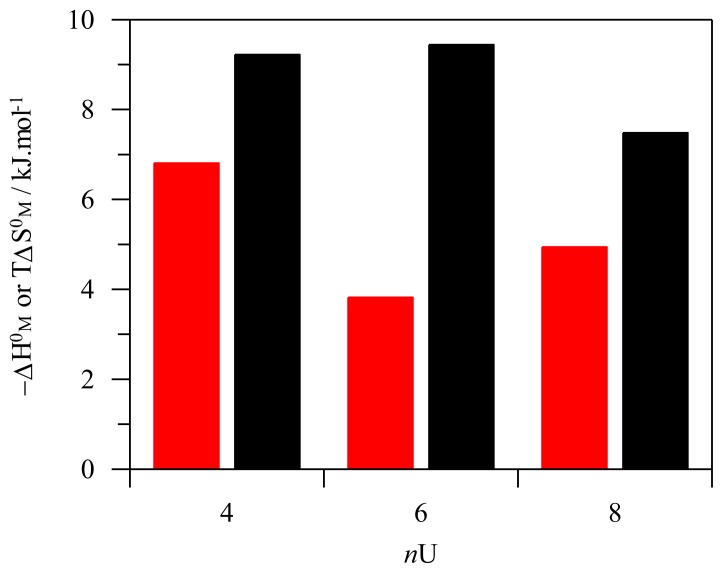
Variation of the enthalpy (red bars) and entropy (black bars) of micellization with the number of monomeric units (*n*U) present in the structure of the amphiphilic *p*-sulfonatocalix[*n*]arenes. All data is given in kJ·mol^−1^ of alkyl chain units.

Since the CMC increases with the number of monomeric units present in the structure of the amphiphile, thus, and according to Equation 1, the Δ*G*_M_ absolute value decreases (become less negative). In the case of Δ*H*_M_, it was found that its absolute value decreases in the order tetramer < octamer < hexamer, while in the case of Δ*S*_M_, the sequence found was tetramer ≈ hexamer > octamer (see Figure 7). The results show that the higher tendency of the tetramer to micellize has both enthalpic and entropic origin, and their interpretation relies in the conformations adopted by the calixarenes below and above the CMC; since the cone conformation is preferred for the formation of globular aggregates, the tetramer benefits from its preorganization in this conformation. On the other hand, both the hexamer and the octamer experiment a structural reorganization upon aggregation, which had an extra energetic cost (in comparison with the tetramer). In the case of the hexamer, this cost is purely enthalpic and can be explained by considering the enthalpy necessary to disrupt the pseudo-1,2,3-alternate conformation adopted by this calixarene below the CMC. On the other hand, for the octamer, the energetic cost is both enthalpic and entropic. In this case, the enthalpic term is lower than that observed for the hexamer, because this specie is much more flexible, and thus, less enthalpy is required to change it to the cone conformation. However, the advantage of the flexibility in the enthalpy balance is paid in entropy. While both the hexamer and the tetramer present similar entropies of micellization, the octamer presents a decrease of almost 2 kJ·mol^−1^. This tendency is again compatible with the higher conformational flexibility of the free unimers that lose several degrees of freedom, due to higher organization and loss of flexibility, when the cone conformation is adopted in the aggregates.

**Figure 8:**
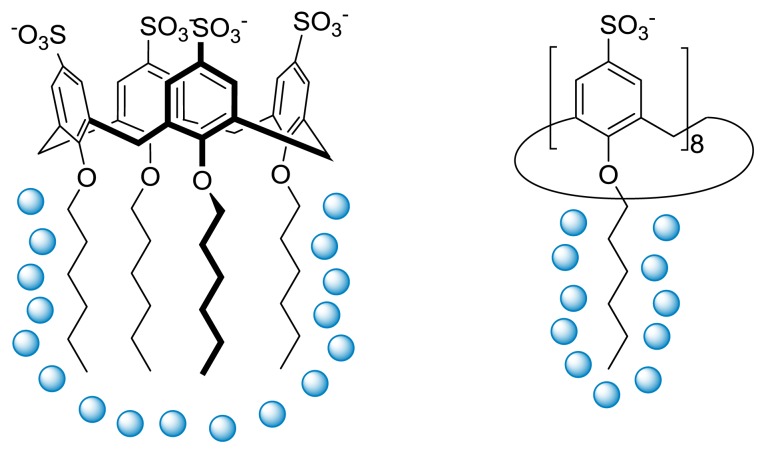
Conformation-dependent hydration of the alkyl chains of *p*-sulfonatocalix[*n*]arenes.

It was also interesting to compare the change in heat capacity of micellization (Δ*Cp*_M_) obtained for the different calixarene-based surfactants. These data can be directly obtained from the variations of the Δ*H*_M_ with the temperature and can provide information about the extent of hydrophobic area that is dehydrated upon micellization. The lower (more negative) value obtained for the octamer seems to indicate that the alkyl chains are individually hydrated, which is compatible with the high degree of conformational mobility associated with this compound. On the other hand, both the hexamer and the tetramer present higher values, suggesting that the alkyl chain might share the same hydration shell. Conversely, these results are compatible with the existence of intramolecular dispersion interactions between the alkyl chains of these surfactants in their unimeric state (Figure 8).

Additionally, the ITC data also allowed the direct determination of the aggregation number (*N*). The results were shown to vary between nine (for **3e** at 288 K) and 23 (for **3e** at 308–328 K). It must be noted that the *N* values obtained for **3e** are more compatible with that obtained by Shinkai *et al.* from the solubilization test of orange OT (19) than that determined by light scattering (6) and small-angle X-ray scattering experiments (7) [39,40]. Another interesting feature of these values is that in the case of **3e***N* seems to vary gradually with the temperature, indicating that structure of the micellar aggregates is temperature-dependent. This is in line with the odd dependence of the CMC with the temperature and deserves to be further studied.

## 3. *p*-Sulfonatocalixarene-Based Supramolecular Amphiphiles

**Figure 9:**
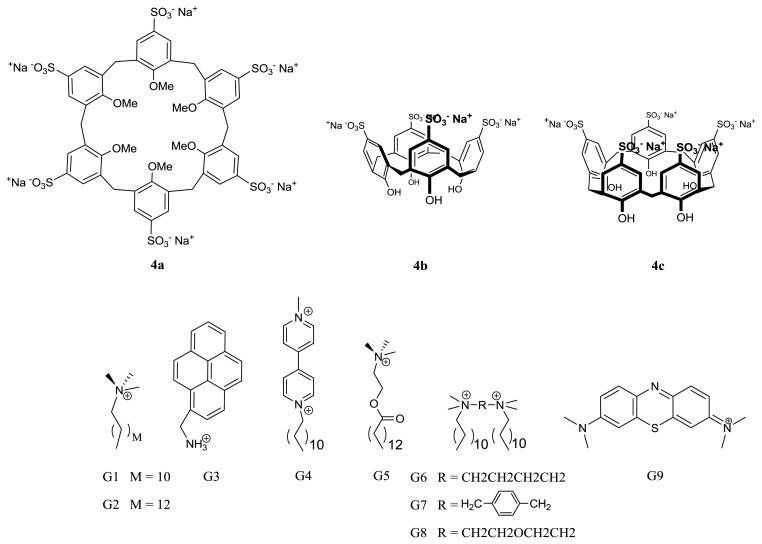
Structures of *p*-sulfonatocalixarene hosts and cationic guests used for the assembly of supramolecular amphiphiles.

**Figure 10:**
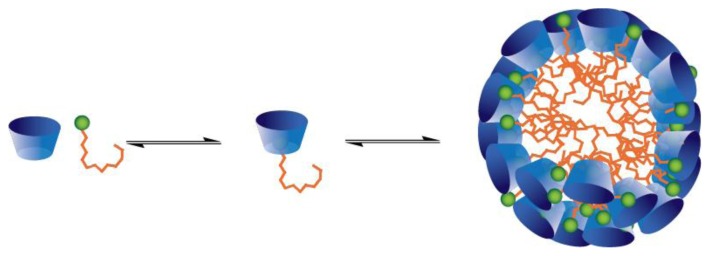
Promoted aggregation of alkyltrimethylammonium surfactants through the formation of host-guest complexes with calix [6]arene **4a** (Reprinted with permission from ref. [66]. Copyright 2012, American Chemical Society).

Among the various water soluble calixarenes, sulfonatocalixarenes have been more frequently employed in the last few years in building self-assembled nanostructures based on noncovalent interactions (Figure 9). Our group reported for the first time the formation of micelles from the complexation of a single chain surfactant with calixarene derivatives in aqueous solution [65]. In that work, besides the formation of an expected host-guest complex between the alkyltrimethylammonium cation (**G1**) and the hexamethylated *p*-sulfonatocalix [6]arene (**4a**), the onset for the formation of micellar aggregates was observed to occur at concentration 70-fold below the CMC of the pure surfactant, suggesting that the calixarene promotes the formation of micelles. The system was characterized by a wide variety of techniques, including NMR, surface tension and dynamic light scattering. The results indicate that for concentrations below the critical aggregation concentration (CAC), the calixarene **4a** forms a discrete 1:1 host-guest complex with **G1** that further aggregates into mixed micelles upon increasing the concentration of surfactant **G1** (Figure 10). Saturation transfer difference NMR experiments point out that the ion-dipole interactions established between the OMe pendant groups of the host and the NMe_3_^+^ group of the surfactant play a significant role in stabilization of supramolecular amphiphile [66].

On the other hand, when the single chain surfactant is in the presence of a smaller and preorganized calixarene, such as *p*-sulfonatocalix [4]arene (**4b**), the formation of vesicles in aqueous solution was observed [67]. The unilamellar vesicles built from the complex formed between **G2** and host **4b** have, after sonication, a diameter of around 120 nm with the potential of being stored by lyophilization and then rehydrated without significant change in size or shape. While there are several host-guest complexes made from **4b** and cationic species that display a bilayer-type arrangement in the solid state, the structure and composition of the vesicle bilayer remains unknown [25]. The critical packing parameter (CPP) is frequently invoked to predict the shape and type of aggregates formed from amphiphilic species [68–70]. The CPP can be defined as *V*_H_/*l*_c_*a*_0_, where *V*_H_ is the volume occupied by the hydrophobic groups in the micellar core, *l*_c_ is its length and *a*_0_ is the cross-sectional area occupied by the hydrophilic group at the micelle-solution interface. This parameter is a measure of the curvature at the aggregate-solution interface, and it can be used to predict the shape of the micelles based in the structure of the surfactant. For example, spherical micelles have CPP values below 1/3, rod-like micelles between 1/3 and 1/2 and lamellar structures are usually formed when CPP values are between 1/2 and 1 [71,72]. On the basis of these considerations, it is important to remark that the formation of vesicles cannot be explained by a 1:1 complex, since, in this case, the *a*_0_ parameter is expected to substantially increase, while *V*_H_ and *l*_c_ are subject to smaller variations. This will lead to a decrease in the CPP of the supramolecular amphiphile in comparison with the free surfactant guest, and thus, micelles should be formed. In order to explain the formation of vesicles, a high order 1:*n* calixarene:surfactant complex must be taken into account. In this case, *V*_H_ is multiplied by *n*, and thus, the CPP can conveniently increase to values higher than 1/2. Moreover, the charge neutralization of high-order complexes can also contribute to reduce the curvature of the interface between the hydrophilic and hydrophobic domains and, consequently, favor the formation of vesicles. In addition to the fact that the CPP model can fail in the prediction of vesicular structures for conventional surfactants, its application to SCn-based supra-amphiphiles presents serious limitations, due to the fact that the structure and composition of the complex or complexes are not currently known [70].

**Figure 11:**
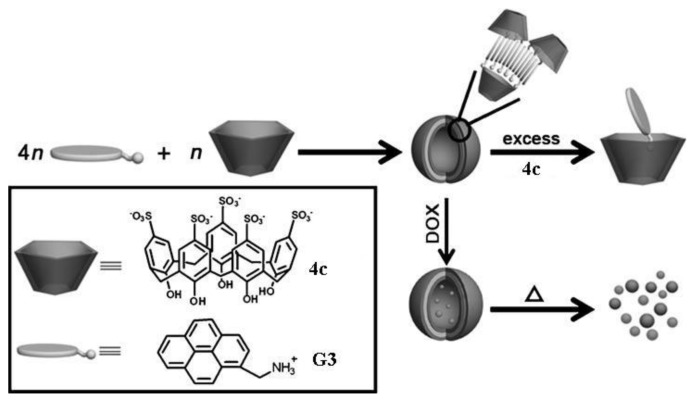
Formation of supramolecular binary vesicles made from pyrene derivative **G3** and calixarene **4c** (Reprinted with permission from ref. [75]. Copyright 2010 Wiley-VCH).

In the last two years, Liu and coworkers had made important contributions to the study of supramolecular binary vesicles assembled from host-guest complexes. In their published works, *p*-sulfonatocalixarene **4b** and **4c** were used as hosts, while molecules **G3**–**G8** were employed as guest [73–76]. In the first report, *p*-sulfonatocalix [5]arene (**4c**) and 1-pyrenemethylaminium (**G3**) were used to construct the vesicular assembly. The aggregation process can be followed by monitoring the polarity-dependent fluorescence spectra of the pyrene moiety. By performing this task in absence and presence of the calixarene, the authors determined the best molar fraction of the complex with the tendency toward self-aggregation [75]. The best mixing ratio for amphiphilic aggregation is when the calixarene **4c** is in the presence of four **G3** molecules, otherwise increasing the amount of calixarene leads to the formation of 1:1 inclusion complexes, accompanied by the disassembly of the amphiphilic aggregation (Figure 11). The authors proposed a model for the unilamellar membrane, where the pyrene segments are stacked together and the inner- and outer-layer surfaces consist of hydrophilic phenolic hydroxyl groups of **4c** exposed to water [75]. This model is also depicted in Figure 11.

Further development of this system was described in posterior reports. In the case of **4b** and 1-meth-yl-1′-dodecyl-4,4′-bipyridinium (**G4**), a value for **4b**/**G4** molar ratio of 0.5 was found to be the ideal for amphiphilic aggregation, while in the case of **4b** and gemini surfactants (**G6**–**G8**), a value of 0.4 was determined [74,76]. On the other hand, for the binary system composed by **4b** and myristoylcholine (**G5**), the results indicate that a molar ratio of 0.1 should be used [73]. Similarly to the first report, increasing the concentration of calixarene affords a simple 1:1 inclusion complex between **4b** and the guests. The CAC’s were also measured for the three guests in the presence and absence of calixarene **4b**. The most interesting feature was observed for the guests **G4** and **G6**–**G8** with a decrease of almost *ca.* 1000 in the CAC value after complexation by the host. For **G5** and **G3**, a decrease of 100- and 3-times were observed, respectively. It should be noted that **4b**, as well as **4c**, do not have any tendency to self-aggregation [77]. To obtain further evidences on the self-assembled morphology of this type of aggregate, the same common techniques in the study of other classes of vesicles are employed. Dynamic laser scattering (DLS), cryo-Transmission Electron Microscopy (Cryo-TEM), Scanning Electron Microscopy (SEM) and Atomic Force Microscopy (AFM) represent the first evidence of vesicle formation in the systems, undoubtedly proving the presence of an inner hollow sphere surrounded by a bilayer or a multilayer [78].

The self-assembly process is defined in the literature as the spontaneous organization of individual components into an ordered structure [79]. The possible control over the characteristics of the components of these aggregates, as well as the interactions among them, makes fundamental investigations in this kind of structures especially tractable. Frequently, stimuli, such as pH, temperature, voltage, redox and light, are employed as a way to have control over the structure and enhance the possibility of using this kind of aggregate as a potential delivery model for special substrates [80–87]. The group of Liu and coworkers also study responsiveness of the binary supramolecular vesicles upon external stimuli. The aggregates formed by **4c** and **G3** can be assembled and disassembled with the variation of temperature [75]. The reason for these behaviors can be explained based on taking into account the type of complexation of the guest by the host and also by the π-π stacking of the guest, which are both enthalpy-driven and, therefore, can be weakened upon heating [17,88,89]. The process of disassembly of the aggregate was also confirmed by loading the vesicles with the doxorubicin hydrochloride (DOX) fluorescence dye, which is released upon increase of the temperature (Figure 11).

Regarding the binary vesicles of **4b** and **G4**, the structure can respond to multiple external stimuli, such as temperature, host-guest competitive binding and redox [74]. Like in the previous report, by increasing the temperature, the aggregates can be disassembled into free **4b**, free **G4** and complex **4b:G4**. Control of the architecture can also be achieved by adding another macrocycle to the aqueous solution where vesicles are present. Through the addition of α-, β- and γ*-*cyclodextrins, the disassembly of the vesicles was confirmed by the absence of Tyndall effect of the solution and also by DLS experiments. This behavior can be explained, since the alkyl chain moiety of the **G4** can form complexes with cyclodextrin and then disrupt the host-guest complex that forms vesicles. Additional experiments to have control of the aggregates were obtained by adding hydrazine and, therefore, changing the state of the guest. The size of the vesicles is smaller when **G4** is reduced from the dication state to the cation, and finally, the aggregate is disrupted in the neutral form of the guest. The self-assembly vesicles constructed with **4b** and **G5** can be controlled by cholinesterase, which induces the disassembly of the vesicles, since it is a specific enzyme to cleave the guest molecules [73]. With this approach, the authors were able to release the entrapped drugs inside the vesicles.

Colloidal aggregates were also obtained from the binary complex formed between methylene blue (**G9**) and SCn. The presence of such aggregates was demonstrated by resonance Rayleigh scattering measurements, where the presence of a band around 560 nm appears in the spectra of **G9** in the presence of three different hosts [90]. The most fascinating aspect of these supramolecular amphiphiles is that two components can self-assemble into higher-order structures, which exhibit particular properties and functions that the individual components cannot achieve.

## 4. Conclusions and Outlook

The work done so far on the study of *p*-sulfonatocalix[*n*]arenes-based surfactants, synthesized both from a covalent or supramolecular approach, demonstrates the high potential of these compounds in the fields of surfactant or colloidal chemistry. When compared with traditional single chain surfactants, covalent *p*-sulfonatocalix[n]arene-based surfactants present a higher tendency to self-aggregate, a property that might be interesting for practical applications and special structural features, such as variable flexibility and conformational reorganization ability, which makes these compounds of special interest to study the effects of these properties on the aggregation of amphiphilic molecules. It is worth noting that these issues are very difficult to address in the case of more conventional amphiphiles. Despite all the efforts, the study of the aggregation properties of these compounds still is in its infancy and there are several basic issues that remain unexplored, such as their interfacial properties and the aggregation behavior of derivatives bearing long alkyl chains.

The field of *p*-sulfonatocalix[*n*]arene-based supramolecular amphiphiles was recently introduced, and it has already provided promising results and potential applications in the fields of smart materials and controlled delivery systems. The basic idea relies in the utilization of the recognition abilities of *p*-sulfonatocalix[n]arenes to form supramolecular complexes with (improved) amphiphilic properties. Due to the inherent reversibility of the non-covalent interactions, the assembly/disassembly process can be conveniently controlled with appropriate stimuli. While these principles had already been demonstrated, questions related to the mechanism of the hierarchical aggregation processes, structure of the supramolecular complex(es) that undergo aggregation and detailed composition of the aggregates have not been answered in order to improve these systems and rationally design more sophisticated ones.
